# Supplementary material for: The exquisitely preserved integument of Psittacosaurus and the scaly skin of ceratopsian dinosaurs
Source: Commun Biol. 2022 Aug 12;5:809. doi: 10.1038/s42003-022-03749-3 (PMC9374759; doi:10.1038/s42003-022-03749-3)
Supplement: Supplementary file 2 — Supplementary Information [file 42003_2022_3749_MOESM2_ESM.pdf]

ELECTRONIC SUPPLEMENTARY INFORMATION

for

The Exquisitely Preserved Integument of *Psittacosaurus* and the Scaly Skin of Ceratopsian Dinosaurs

Phil R. Bell1*†, Christophe Hendrickx2*†, Michael Pittman*, Thomas G. Kaye, Gerald Mayr

*Corresponding author emails: pbell23@une.edu.au; christophendrickx@gmail.com; mpittman@cuhk.edu.hk

†Authors contributed equally to this work.

This file includes:

Supplementary Note 1 2

Supplementary Table 1 3

Supplementary Table 2 4

Supplementary References 8

Supplementary Note 1

AMNH FARB American Museum of Natural History, fossil amphibian, reptile, and bird collections, New York, USA. **CMN** Canadian Museum of Nature, Ottawa, Quebec, Canada. **FHSM** Sternberg Museum of Natural History, Hays, Kansas, USA. **GMC** Geological Museum of China, Beijing, China. **HMNS** Houston Museum of Natural Science, Houston, Texas, USA. **IVPP** Institute of Vertebrate Paleontology and Paleoanthropology, Beijing, China. **LPM** Liaoning Paleontological Museum, Shenyang, Liaoning Province, China. **MN** Museu Nacional, Universidade Federal do Rio de Janeiro, Rio de Janeiro, Brazil. **MOR** Museum of the Rockies, Bozeman, Montana, USA. **MV** Palaeontological Museum (Nanjing Institute of Geology and Palaeontology), Nanjing, Jiangsu Province, China. **PKUP** Peking University Vertebrate Paleontology, Beijing, China. **SMF** Senckenberg Research Institute and Natural History Museum Frankfurt, Hesse, Germany. **STM** Shandong Tianyu Museum of Nature, Pingyi, Shandong Province, China. **SUP** Shenandoah University Collection, Winchester, Virginia, USA. **UALVP** University of Alberta vertebrate palaeontology collection, Edmonton, Alberta, Canada. **YFM** Yizou Fossil Museum, Yixian County, Liaoning Province, China.

Supplementary Table 1.

Scale morphology in the ceratopsian dinosaur *Psittacosaurus* sp. (SMF R 4970).

Body region	Basement scales		Feature scales	
	**Morphology**	**Diameter (mm)**	**Morphology**	**Diameter (mm)**
**Head**	Oval to subcircular	0.5-3	Absent	-
**Neck**	Lenticular, elliptical to rounded polygonal	0.7-2	Absent	-
**Shoulder**	Polygonal or rounded-polygonal	1-1.4	Cylindrical or truncated-cone shaped, stripped	6.7-9.8 mm
**Forelimbs**	Polygonal or rounded-polygonal; hexagram arrangement	1-1.5	Absent	-
**Manus**	Reticulate	0.5	Absent	-
**Flank**	Diamond-shaped	1.1-2.8	Circular to irregular	3-4 mm
**Abdomen**	Rectangular	1.1-2.2	Absent	-
**Hindlimbs**	Diamond-shaped; hexagram pattern	0.5-3.1	Absent	-
**Tarsus**	Reticulate	0.9-1.8	Absent	-
**Cloaca and ischial callosity**	lenticular, sub-triangular, rounded-rectangular or hexagonal	3-6.8	Absent	-
**Tail**	rounded-quadrangular	1.3-6.8	Quadrangular	>8 mm

Supplementary Table 2.

Skin distribution in ceratopsian dinosaurs.

Genus	Specimen	Locality	Formation and age	Body region	Type of integument	Author(s)
Psittacosaurus mongoliensis	AMNH FARB 6260	Red Mesa, Artsa Bogdo, Oshih Basin, Mongolia	Öösh Formation; ?Hauterivian	Pes	Reticulate scales	[1]
Psittacosaurus sp.	YFM-R001	Sihetun Village, Beipiao City, western Liaoning Province, China	Jianshangou Bed, Yixian Formation; Upper Barremian - Lower Aptian	Shoulder (humerus), forelimb (scapula), hindlimb (femur)	Hexagonal and triangular basement scales	[2–4]
Psittacosaurus sp.	MV 53	Nanjing, Liaoning Province, China	Jehol Biota, Yixian Formation; Upper Barremian - Lower Aptian	Flank	Collagen fibers	[5,6]
Psittacosaurus houi (= *P. lujiatunensis)*	PKUP V1050	China	NA; Lower Cretaceous	Hips	Skin	[7]
Psittacosaurus houi (= *P. lujiatunensis)*	PKUP V1051	China	NA; Lower Cretaceous	Tail + ?	Skin	[7]

Psittacosaurus sp.	SMF R 497	Jehol deposits of the Liaoning Province; most likely from the Sihetun locality, Beipiao County, Liaoning Province, China	Most likely Jianshangou Bed, Yixian Formation; Barremian - Aptian	Head, neck, shoulder, forelimbs, manus, flank, abdomen, hips, hindlimbs, tail	Quadrangular, rectangular, polygonal, irregular basement scales; reticulate scales; rounded and quadrangular feature scales	8–12
Possible Psittacosauridae	GMC LL2001-01	Dawangzhangzi Village, Lingyuan, Liaoning Province, China	Jehol Biota, Yixian Formation; Upper Barremian - Lower Aptian	Neck and/or shoulder, forelimb, hindlimbs (carpals)	Rounded, polygonal, triangular basement scales; Rounded feature scales	13
Protoceratops andrewsi	AMNH FARB 6418	Shabarakh Usu, Omnogov Province, Mongolia	Bayn Dzak Member; Djadochta Formation; Upper Campanian	Head	Possible pebbly basement scales	14

Centrosaurus apertus	AMNH FARB 5351	RTMP Quarry 105, Sand Creek; 100 feet below top of beds, north fork of Sand Creek, 12 miles below Steveville, Red Deer River, Alberta, Canada	Dinosaur Park Formation; Upper Campanian	Flank, abdomen?	Polygonal basement scales; dermal plates	[15]
Centrosaurus apertus	AMNH FARB 5427	north fork, Sand Creek, Alberta, right bank 50 feet above river Red Deer River, Canada	Oldman Formation; Campanian	Flank	Polygonal basement scales; rounded feature scales	[15,16]
Centrosaurus sp.	TMP 1986.018.0097	Dinosaur Provincial Park, *Centrosaurus* bonebed	Dinosaur Park Formation	Tail	Polygonal basement scales	/
Chasmosaurus belli	CMN 2245	Chasmosaurus type, Berry Creek, Red Deer River, Alberta, Canada	Dinosaur Park Formation; Upper Campanian	Hips	Polygonal basement scales; rounded feature scales	[16–18]
Chasmosaurus belli	UALVP 52613	Quarry Q255 in northeastern part of Dinosaur Provincial Park, Alberta, Canada	Dinosaur Park Formation; Upper Campanian	Flank, hindlimbs	Polygonal basement scales	[19]
Chasmosaurus	FHSM VP-	Near Steveville,	Belly River	Unknown	Polygonal	/

sp.	117	Alberta, Canada	Formation		basement scales; possible subcircular feature scales	
Nasutoceratops titusi	UMNH VP 16800	Grand Staircase–Escalante National Monument, southern Utah, USA	Kaiparowits Formation; Upper Campanian	Shoulder, forelimb	Triangular basement scales; oval to subcircular and hexagonal feature scales	[20,21]
Triceratops horridus ("Lane")	HMNS PV.1506	Zerbst Ranch, Converse County, Niobrara (county), Wyoming, USA	Lance Formation; Maastrichtian	Neck, forelimb, flank, abdomen	Polygonal basement scales; Polygonal feature scales with nipple like structures	[22]
Triceratops prosus	CMN FV 56508	north side, Frenchman River, Eastend, Saskatchewan, Canada	Frenchman Formation; Maastrichtian	Head	Polygonal basement scales	
Triceratops sp.	?	Jordan, Garfield	Hell Creek	?	NA	[23]

		County, Montana, USA	Formation; Maastrichtia n			
Triceratops sp.	?	Montana, USA	Late Cretaceous	Head	?	24

Supplementary References

1. Sereno, P. C. The ornithischian dinosaur *Psittacosaurus* from the Lower Cretaceous of Asia and the relationships of the Ceratopsia. (Columbia University, 1987).
2. Ji, S. A. & Bo, H. C. Discovery of the psittacosaurid skin impressions and its significance. *Geological Review* **44**, 603–606 (1998).
3. Ji, S. Initial report of fossil psittacosaurid skin impression from the uppermost Jurassic of Sihetun, northeastern China. *Earth Science (Chikyu Kagaku)* **53**, 314–316 (1999).
4. Glut, D. F. *Dinosaurs: The Encyclopedia, Supplement 2*. (2002).
5. Feduccia, A., Lingham-Soliar, T. & Hinchliffe, J. R. Do feathered dinosaurs exist? Testing the hypothesis on neontological and paleontological evidence. *Journal of Morphology* **266**, 125–166 (2005).
6. Lingham-Soliar, T. A unique cross section through the skin of the dinosaur *Psittacosaurus* from China showing a complex fibre architecture. *Proceedings of the Royal Society B: Biological Sciences* **275**, 775–780 (2008).
7. Li, Q. *et al.* Melanosome evolution indicates a key physiological shift within feathered dinosaurs. *Nature* **507**, 350–353 (2014).
8. Mayr, G., Peters, S. D., Plodowski, G. & Vogel, O. Bristle-like integumentary structures at the tail of the horned dinosaur *Psittacosaurus*. *Naturwissenschaften* **89**, 361–365 (2002).

9. Mayr, G., Pittman, M., Saitta, E., Kaye, T. G. & Vinther, J. Structure and homology of *Psittacosaurus* tail bristles. *Palaeontology* **59**, 793–802 (2016).
10. Lingham-Soliar, T. & Plodowski, G. The integument of *Psittacosaurus* from Liaoning Province, China: taphonomy, epidermal patterns and color of a ceratopsian dinosaur. *Naturwissenschaften* **97**, 479–486 (2010).
11. Vinther, J. *et al.* 3D camouflage in an ornithischian dinosaur. *Current Biology* **26**, 2456–2462 (2016).
12. Vinther, J., Nicholls, R. & Kelly, D. A. A cloacal opening in a non-avian dinosaur. *Current Biology* **31**, R182–R183 (2021).
13. Ji, S. Preliminary report on the dinosaurian skin impressions from the Yixian Formation of Lingyuan, Liaoning. *Geological Review* **50**, 170–174 (2004).
14. Brown, B. & Schlaikjer, E. M. The structure and relationships of *Protoceratops*. *Annals of the New York Academy of Sciences* **40**, 133–266 (1940).
15. Brown, B. A complete skeleton of the horned dinosaur *Monoclonius*, and description of a second skeleton showing skin impressions. *Bulletin of the American Museum of Natural History* **37**, 298–306 (1917).
16. Lull, R. S. A revision of the Ceratopsia or horned dinosaurs. *Peabody Museum of Natural History Bulletin* **3**, 1–175 (1933).
17. Lambe, L. M. On the fore-limb of a carnivorous dinosaur from the Belly River Formation of Alberta, and a new genus of Ceratopsia from the same horizon, with remarks on the integument of some Cretaceous herbivorous dinosaurs. *The Ottawa Naturalist* **27**, 129–135 (1914).
18. Sternberg, C. M. Integument of *Chasmosaurus belli*. *Canadian Field Naturalist* **39**, 108–110 (1925).

19. Currie, P. J., Holmes, R. B., Ryan, M. J. & Coy, C. A juvenile chasmosaurine ceratopsid (Dinosauria, Ornithischia) from the Dinosaur Park Formation, Alberta, Canada. *Journal of Vertebrate Paleontology* **36**, e1048348 (2016).

20. Lund, E. K. *Nasutuceratops titusi*, a new basal centrosaurine dinosaur (Ornithischia: Ceratopsidae) from the Upper Cretaceous Kaiparowits Formation, southern Utah. (The University of Utah, 2010).

21. Lund, E. K., Sampson, S. D. & Loewen, M. A. *Nasutoceratops titusi* (Ornithischia, Ceratopsidae), a basal centrosaurine ceratopsid from the Kaiparowits Formation, southern Utah. *Journal of Vertebrate Paleontology* **36**, e1054936 (2016).

22. Larson, P., Larson, M., Ott, C. & Robert, B. Skinning a *Triceratops*. in *Journal of Vertebrate Paleontology* vol. 27 104A (2007).

23. Happ, J. W. & Morrow, C. M. Bone modification of subadult *Triceratops* (Dinosauria: Ceratopsidae) by crocodilian and theropod dining. in *Abstract of Papers. Fifty-Seventh Annual Meeting*, *Society of Vertebrate Paleontology* vol. 17 (3) 51A (Journal of Vertebrate Paleontology, 1997).

24. Lessem, D. Skinning the Dinosaur. *Discover* **10**, 38–44 (1989).
