# Supplementary material for: The exquisitely preserved integument of Psittacosaurus and the scaly skin of ceratopsian dinosaurs
Source: Commun Biol. 2022 Aug 12;5:809. doi: 10.1038/s42003-022-03749-3 (PMC9374759; doi:10.1038/s42003-022-03749-3)
Supplement: Supplementary file 3 — Description of Additional Supplementary Files [file 42003_2022_3749_MOESM3_ESM.pdf]

Description of Additional Supplementary Files

File name: Supplementary Data
Description: Data on scale diameters in SMF R 4970, cloacal morphologies in Reptilia, skin specimens in Ceratopsia, horn dimension in various amniotes, and basement scales vs. body mass in Dinosauria (Excel file).
